# Proctalgia fugax with dysthymia

**DOI:** 10.4103/0019-5545.31606

**Published:** 2006

**Authors:** Gurvinder Pal Singh

**Affiliations:** * Senior Lecturer Department of Psychiatry, Government Medical College and Hospital, Sector 32, Chandigarh

## Abstract

A rare case of proctalgia fugax with dysthymia which was successfully treated with dothiepin and other psychological interventions.

## INTRODUCTION

Proctalgia fugax is characterized by uncomfortable rectal pain that appears suddenly at night.^1^ The diagnosis and treatment of proctalgia fugax is usually a challenge.^2^ Patients with this condition are not usually referred to the psychiatric services, even though they have demonstrable psychiatric morbidity.^3^ A case of proctalgia fugax with dysthymia is described.

## THE CASE

SK, a 61-year-old retired government employee, was referred for psychiatric consultation by a laparoscopic surgeon. He complained of severe, intractable anorectal pain for the past 3 years. The pain was severe, abrupt, sharp or gripping in nature and woke him up at night. The pain was localized, lasted for several seconds to 2–3 minutes and occurred 4–5 times a week. He was advised a warm bath or ice pack by a private practitioner but his symptoms remained unchanged. After 3 months, his family members consulted a surgeon and gastroenterologist. His general physical and systemic examination, as well as all routine and special investigations (ultrasonography and CT scan abdomen, proctosigmo-idoscopy and rectal biopsy) were normal and he was diagnosed as having proctalgia fugax. He was given a course of tetanus prophylaxis, antibiotics and nitroglycerin ointment for local application. He stopped treatment because there was no improvement.

On psychiatric evaluation, the patient was anxious and highly strung, and gave no history of psychiatric illness prior to the development of proctalgia fugax. He was found to have chronic low mood and fulfilled all the ICD-10 criteria for dysthymia.^4^ He was prescribed dothiepin 75 mg/day, which was gradually increased to a maximum of 150 mg/day. The patient was also advised psychological interventions including psychoeducation and Jacobson progressive muscle relaxation therapy. He came regularly for follow-up thrice a week and compliance with treatment was good. Family therapy sessions were also planned. Within the first week the patient reported improvement in his symptoms. His sleep improved and the severity, frequency and duration of rectal pain had diminished. In the next 3 weeks his sadness of mood and other depressive phenomenology also improved. He has been asymptomatic for the past 6 months.

## DISCUSSION

This patient presented with symptoms of anorectal pain. All other systemic causes of anorectal pain were ruled out on detailed evaluation. Anorectal and perineal pain can occur in the absence of organic disease.^5^ Proctalgia fugax, also known as doctor's disease, is common, but most patients do not seek psychiatric advice. The aetiology is unknown, but it has been suggested that the condition may be a variation of irritable bowel syndrome, pelvic floor myalgia, and internal anal sphincter spasm.^6^ This disease is considered incurable and many pharmacological treatments have been tried with little success. Renzi and Pescatori^3^ investigated the psychosomatic components of proctalgia and found that depression, anxiety and personality disorders were predominant in these patients.

This is the first case report of proctalgia fugax with dysthymia and its successful treatment with combined therapy (dotheipin and psychological interventions) from the Indian subcontinent. However, further studies are required in a large number of patients. This report highlights the need for more psychiatric research in patients with proctalgia fugax. Better collaboration between the surgical specialist and mental health professional is advocated when dealing with these patients.
